# Cooperation and conflict in host manipulation: interactions among macro-parasites and micro-organisms

**DOI:** 10.3389/fmicb.2014.00248

**Published:** 2014-06-11

**Authors:** Frank Cézilly, Marie-Jeanne Perrot-Minnot, Thierry Rigaud

**Affiliations:** ^1^Equipe Ecologie Evolutive, UMR CNRS 6282 Biogéosciences, Université de BourgogneDijon, France; ^2^Institut Universitaire de FranceStrasbourg, France

**Keywords:** extended phenotype, horizontal transmission, host manipulation, multidimensionality, trophic transmission, vertical transmission

## Abstract

Several parasite species are known to manipulate the phenotype of their hosts in ways that enhance their own transmission. Co-occurrence of manipulative parasites, belonging to the same species or to more than one species, in a single host has been regularly observed. Little is known, however, on interactions between co-occurring manipulative parasites with same or different transmission routes. Several models addressing this problem have provided predictions on how cooperation and conflict between parasites could emerge from multiple infections. Here, we review the empirical evidence in favor of the existence of synergistic or antagonistic interactions between co-occurring parasites, and highlight the neglected role of micro-organisms. We particularly discuss the actual importance of selective forces shaping the evolution of interactions between manipulative parasites in relation to parasite prevalence in natural populations, efficiency in manipulation, and type of transmission (i.e., horizontal versus vertical), and we emphasize the potential for future research.

Several parasite species have evolved the ability to alter the phenotype of their hosts in ways that enhance their own fitness at the expense of that of their hosts (Moore, 2002; Thomas et al., 2005; Lefèvre et al.,, 2009). Such cases of so-called “host manipulation” by parasites are commonly regarded as compelling examples of extended phenotypes (Dawkins, 1982), and can be conveniently grouped in two broad categories depending on the way host fitness is affected. First, parasites may increase mortality risks faced by their host in order to increase their own transmission rate. For instance, increased horizontal transmission has been regularly evidenced in parasites with complex life cycles (such as acanthocephalans, cestodes, or trematodes) that alter the phenotype of their invertebrate intermediate hosts in ways that appear to increase vulnerability to predation by vertebrate final hosts (Bethel and Holmes, 1973; Kaldonski et al., 2007; but see below). Second, parasites may increase their transmission by altering the reproduction of their hosts in different ways. For instance, various vertically transmitted obligate intracellular parasites parasites, such as *Wolbachia* bacteria or microsporidia, have evolved the ability to feminize their hosts, thus increasing their rate of transmission (Terry et al., 2004; Werren et al., 2008; Engelstädter and Hurst, 2009) whereas helminths and over parasites may induce partial or total castration in their hosts (Thomas et al., 1996; Bollache et al., 2002; Jensen et al., 2006), resulting in the reallocation of host resources towards maintenance, and, hence, improved development and survival of the parasites.

Most studies of host manipulation by parasites have considered the case where hosts are infected by only one macroparasite, or a single parasite species (see Moore, 2002 for a review). However, multiple and/or mixed parasite infections have been shown to occur in a large range of host–parasite associations (Graham, 2008; Rigaud et al., 2010; Ferrari and Vavre, 2011; Viney and Graham, 2013), with the possibility that interactions between co-occurring parasites influence the timing and/or the type of phenotypic alterations observed in infected hosts. In particular, manipulative parasites may share a common interest in host manipulation or, alternatively, may compete to take the control of their common host (Lafferty, 1999), particularly when they depend on alternative pathways for successful transmission. So far, the potential for cooperation or conflict between co-occurring parasites has been largely addressed from a theoretical point of view, whereas empirical evidence remains limited. Here we review the literature on cooperation and conflict in host manipulation, discuss the importance of transmission routes and prevalence in shaping interactions between co-occurring parasites, and particularly emphasize the neglected role of micro-organisms.

## HOST MANIPULATION: A CRITICAL INTRODUCTION

Before addressing the relevance of interactions between parasites to the study of host manipulation, it is worth examining the extent and variety of phenotypic alterations brought about by parasites in their hosts, as well as their consequences for both parties in the interaction. Depending on the host-parasite system under consideration, parasite-induced phenotypic alterations can consist of modified appearance, aberrant behavior, disrupted physiology, or a combination of the three (Moore, 2002; Cézilly et al., 2013). As an illustration, we will focus here on hosts infected with either helminths with complex life cycle or microorganisms. A more complete description of the effects of other types of parasites on the phenotype of their hosts can be found in Moore (2002) and Poulin (2007).

### PHENOTYPIC ALTERATIONS CAUSED BY HELMINTHS WITH COMPLEX LIFE CYCLE IN THEIR INTERMEDIATE HOSTS

Many helminth species with a complex life cycle can alter the phenotype of their hosts in different ways. First, they can modify their appearance, with the effect of making them more conspicuous. One famous example corresponds to bird trematodes belonging to the genus *Leucochloridium* infecting snails as intermediate hosts. Transmission of the parasite to its definitive avian hosts is supposedly facilitated by the rhythmic movement of colored sporocyst broodsacs in the ocular tentacles of infected snails. The resemblance of the broodsacs to caterpillars is believed to lure birds and, thus, increase the probability of trophic transmission to the final host (Lewis, 1977). Second, parasites may affect the behavior of their hosts. For instance, snails infected with *Leucochloridium* have been reported to become positively phototactic and to position themselves in places, situated higher in the vegetation, what presumably increases their exposure to bird predators (Wesołowska and Wesołowski, 2014). However, direct evidence for differential predation of birds on infected snails is lacking.

Interestingly, this typical text-book example of host manipulation comes as a cautionary tale, as it perfectly illustrates two important aspects of the study of host manipulation. First, as observed in snails infected with *Leucochloridium*, infection with manipulative parasites most often affects more than one dimension in host phenotypes, even though earlier studies have generally considered the influence of infection on a single trait at a time. Indeed, although multidimensionality in host manipulation has received attention only recently (Cézilly and Perrot-Minnot, 2005, 2010; Thomas et al., 2010), it seems to be the rule rather than the exception (Cézilly et al., 2013). Second, most studies have concluded that manipulative parasites with complex life cycle benefit from inducing phenotypic changes in their hosts in terms of increased trophic transmission without necessarily proving it. The mere observation that parasite-induced phenotypic alterations show “signs of purposive design” (Poulin, 1995) and are associated with increased susceptibility of infected intermediate hosts to predation by final host is actually no evidence for a causal relationship between the two phenomena (Cézilly et al., 2010), contrary to what has been commonly inferred in the study of host manipulation by parasites, as shown by studies of acanthocephalan parasites and their amphipod hosts.

Amphipod species (crustaceans) are regularly exploited as intermediate hosts by fish and bird acanthocephalans. In several of them, mature cystacanths (the infective stage to the definitive host) show typical carotenoid-based orange colorations (see Gaillard et al., 2004) which are visible through the translucid cuticle of their crustacean intermediate hosts, and, presumably, make them more conspicuous to predators. In addition, infection with such parasites generally alters the natural negative phototaxis of their hosts, such that infected individuals become indifferent or attracted to light, whereas uninfected ones are strongly repulsed by it (Bethel and Holmes, 1973; Cézilly et al., 2000). However, the phenotypic effect of infection with acanthocephalans goes well beyond altering appearance and reaction to light as, for instance, no less than 15 different phenotypic alterations have been reported in the crustacean amphipod *Gammarus pulex* infected by the acanthocephalan parasite *Pomphorhynchus laevis*, including several physiological effects such as reduced oxygen consumption, decreased immunocompetence, increased brain serotonergic activity, and increased glycogen levels (Cézilly et al., 2013). Besides, predation experiments have shown that amphipods infected by fish acanthocephalans are more vulnerable to fish predation than uninfected ones (Bakker et al., 1997; Kaldonski et al., 2007), suggesting that at least some of the phenotypic alterations observed in infected hosts might be adaptive for the parasite. Accordingly, following a series of experiments, Bakker et al. (1997) concluded that both altered host appearance and phototactic behavior were responsible for the increased vulnerability of *G. pulex* infected with the fish acanthocephalan *Pomphorhynchus laevis* to predation by fish, thus providing supposedly firm evidence for the host-manipulation hypothesis. However, more recent studies have shown that this conclusion was erroneous. Using realistic painted mimics, Kaldonski et al. (2009) showed that cystacanths color actually plays no causal role in the increased susceptibility of infected amphipods to fish predation. And nor does altered phototaxis, as evidenced by Perrot-Minnot et al. (2012), through resorting to both phenotypic engineering and predation experiments under contrasted light intensities. One possibility, that remains so far untested, is that the increased vulnerability of infected hosts is due to a combination of phenotypic alterations rather than a single one, hence the importance of addressing multidimensionality in manipulation. Alternatively, infected amphipods might simply be less vigorous than uninfected ones and, hence, less able to escape from predators (Cézilly et al., 2010). Whatever the correct answer is, the investigation of the adaptive consequences of phenotypic alterations induced by parasites with trophic transmission clearly deserves further development.

Helminths with complex life-cycles are also known to alter the reproductive biology of their hosts through reducing male competitiveness and inclination to pair (Zohar and Holmes, 1998; Bollache et al., 2001), or through decreasing female fecundity through partial or total castration (Poulton and Thompson, 1987; Bollache et al., 2002). The impact of helminth parasites on the reproductive physiology of their intermediate hosts seems to be a direct consequence of the energetic cost of parasitism. For instance, in the freshwater isopod intermediate host *Ceacidotea communis* infected by the acanthocephalan parasite *Acanthocephalus tehlequahensis*, infected individuals have been found to allocate about 21% of their net production energy to parasite growth, while allocating zero energy to reproduction (Lettini and Sukhdeo, 2010).

So far, to the best of our knowledge, the effect of helminth parasites on the reproductive biology of their intermediate hosts has been investigated independently of their manipulative effects on other phenotypic dimensions. An important question, however, in the study of host manipulation by parasites is whether those different alterations are independent of each other from a mechanistic point of view, or are linked by common physiological mechanisms of dysfunction (Cézilly and Perrot-Minnot, 2010).

### MICRO-ORGANISMS ASSOCIATED WITH HOST PHENOTYPIC CHANGES

A large variety of micro-organisms, ranging from viruses to protozoa, has been shown to modify their host phenotype, and these phenomena have often been interpreted as cases of host manipulation. Two broad categories can be distinguished. The first involves microparasites modifying host reproduction, for which numerous reviews are available (O’Neill et al., 1997; Duron et al., 2008; Engelstädter and Hurst, 2009). A now classical example of such microorganisms are *Wolbachia* bacteria (O’Neill et al., 1997; Werren et al., 2008), but recent studies evidenced a large spectrum of microorganisms inducing similar effects, probably showing evolutionary convergence (e.g., Terry et al., 2004; Duron et al., 2008; Ferrari and Vavre, 2011) These micro-organisms all show a common trait: they are vertically transmitted, mostly *trans*-ovarially. They may increase the proportion of their transmitting female hosts through inducing sex-reversal in males, or parthenogenesis, or male-killing (Bandi et al., 2001). They may also prevent uninfected individuals or individuals infected with a different strain from reproducing, through inducing cytoplasmic incompatibility (Stouthamer et al., 1999; Perrot-Minnot et al., 1996). Such alterations of host reproductive biology appear to be obligatory for the maintenance of these micro-organisms inside their host, and often favor their spread and fixation. In these cases, the adaptive nature of these reproductive changes for parasite transmission is doubtless.

The second category of manipulative microparasites is those inducing changes in host behavior, as those evidenced for several macro-parasites (see above). There is a growing body of evidence for these changes, encompassing parasites with complex life-cycle and either trophic or vector-borne transmission, and parasites with direct life-cycle (reviews in Lefèvre et al., 2006, 2009; Ezenwa et al., 2012; van Houte et al., 2013). Most of them, if not all, have been interpreted as changes in the host behavior favoring parasite transmission to next hosts, as illustrated in the following examples. In the heterogenous parasite *Toxoplasma gondii*, infected rats become attracted by cat scents, favoring the parasite trophic transmission after ingestion by a feline definitive host (Berdoy et al., 2000; Vyas et al., 2007; but see Worth et al., 2013). Several vector-borne protozoans alter the probing rate, probability of multiple feeding and host choice of their dipteran vector, in ways increasing their chances of getting transmitted (Koella et al., 1998; Lefèvre et al., 2006; Cator et al., 2012). Interestingly, the stage-specific changes in host seeking and attraction induced by *Plasmodium yoelii* infection in its vector *Anopheles stephensi* can be mimicked in uninfected mosquitoes by an immune challenge (Cator et al., 2013). Among vertically transmitted parasites, the filamentous virus (LbFV) infecting the parasitoid *Leptopilina boulardi* is responsible for superparasitism behavior in its female host (Varaldi et al., 2003). Instead of laying their egg in an uninfected *Drosophila* larvae (an optimal laying behavior avoiding superparasitism in this species), *L. boulardi* females infected by LbFV parasitize *Drosophila* larvae already hosting another *Leptopilina* larvae. Experimental data and theoretical modeling showed that by doing so, infected *L. boulardi* mother increase the probability of virus transmission: during intra-*Drosophila* competition, larvae infected by vertical transmission often transmit horizontally the LbFV to other *Leptopilina* larvae, increasing the probability of parasite maintenance in host populations (Varaldi et al., 2003; Gandon et al., 2006). Finally, various insect species, when infected by fungi or viruses with direct life-cycle, exhibit abnormal climbing and clinging behavior in the hours preceding their death. The infectious stages of these parasites are released from dead hosts, and the position of hosts above ground or at the top of plants could increase the chances of dissemination (Goulson, 1997; Roy et al., 2006; Andersen et al., 2009).

In addition, recent studies have evidenced a “puppet master” role of microorganisms in what was thought to be alterations in host behavior brought about by macroparasites. Some ladybird species, when parasitized by Braconidae parasitoids, appear to protect the parasitoid pupae from entomophagous predators through adopting a modified behavior (Maure et al., 2013). It has been recently shown in one of these cases that the “body-guard” behavior expressed by “zombie” hosts is actually induced by a virus released by the developing parasitoid larvae inside the host (Dheilly et al., submitted). As in a system of Russian dolls, the vertically transmitted virus seems to favor its own transmission through increasing the survival of its parasitoid host thanks to the protective behavior it induces in the parasitoid’s host. Another form of protective manipulation possibly involving a symbiont of the parasite is aposematism. The entomopathogenic nematode parasite *Heterorhabditis* enhances its survival by inducing in its dead larval moth the production of warning colorations and distasteful chemicals host that deter bird predators (Fenton et al., 2011). The suspected role of *Photorhabdus luminescens*, the mutualist bacterium of the nematode and the actual parasite of the larval moth, in these changes remains to be demonstrated. Such cases of “ménage à trios” illustrate the importance of considering microorganisms as key players in parasitic interactions, with important consequences on the evolution of virulence itself.

It is worth noting, however, that changes in behavior following infection with a microparasite may sometime correspond to a strategy by which hosts eliminate the infection or reduce its costs, as shown by changes in food preferences (leading to self-medication) or behavioral fevers (insects seeking high temperatures unfavorable to parasite growth) (see Perrot-Minnot and Cézilly, 2009, for a review).

## INTERACTIONS BETWEEN CO-OCCURING PARASITES: THEORETICAL CONSIDERATIONS

Most parasites have highly aggregated spatial distributions (Combes, 1991), meaning that parasites of the same species tend to co-occur within a single host more often than by chance. As a consequence, monospecific mixed infections involving at least two strains are common in nature (several references in Read and Taylor, 2001; Choisy and de Roode, 2010; Alizon et al., 2013). They can give rise to intraspecific competitive or cooperative interactions over host exploitation, with important consequences for the evolution of virulence (Read and Taylor, 2001; Buckling and Brockhurst, 2008; Lively, 2009; Choisy and de Roode, 2010; Alizon et al., 2013). In addition, interactions between co-occurring parasites might be mediated by the host immune system (see for instance Ulrich and Schmid-Hempel, 2012; Fairlie-Clarke et al., 2013). However, because little is known about host-mediated competition between manipulative parasites, we will only consider here direct parasite-parasite interactions, although the role of host immunity clearly deserves further attention. In the case of manipulative parasites, co-infection may promote the evolution of cooperation in manipulation at the within-host level if transmission interests are closely aligned among conspecifics. In this case however, conflict or cooperation may still arise on the distribution of metabolic costs of manipulation among co-infecting parasites, i.e., on the relative manipulative effort. In any case, intraspecific interactions should have direct consequences on the individual optimal level and pattern of manipulative effort. As evidenced for parasite establishment, growth, and reproduction, several parameters are expected to impact the individual parasite’s decision on manipulative strategy in a co-infected host, such as relatedness, parasite load, timing of co-infection and host density (i.e., opportunities for transmission). In addition, parasites belonging to different species, and having similar or contrasted transmission routes, can coexist in a single host. Multiple infections, i.e., individual hosts co-infected by different parasite species, are frequent in nature (Rigaud et al., 2010). They have received recent theoretical attention because of their potential effect on the evolution of parasite virulence and/or transmission (Alizon et al., 2013). In the case of manipulative parasites, co-infection may occur between individuals sharing a common interest or opposite ones. Under the assumption that manipulation is costly, the co-occurrence of several parasites within a single host may then lead to synergistic or antagonistic interactions (Brown, 1999; Lafferty, 1999; Vickery and Poulin, 2010).

The simplest situation is when two or more manipulative parasites belong to a single species and, hence, sharing a common transmission strategy, co-occur in the same host. Brown (1999) modeled a situation where the fitness of each individual parasite decreases as its manipulative effort increases (reflecting the cost of manipulation), and increases as a function of the total manipulation effort achieved by all parasites present in the host. Under such circumstances, each individual parasite may lower its own manipulative effort while keeping constant its probability of transmission or, even, increasing it (Brown, 1999; Vickery and Poulin, 2010). However, the extent of cooperation between parasites is highly dependent upon both *n*, the size of the infrapopulation (being zero when *n* is small), and the efficiency of “passive” transmission (i.e., transmission when no manipulation occurs). Besides, an individual’s investment in host’s behavioral manipulation will benefit others in terms of increased transmission chances to the next host, and, as such, manipulative effort directed towards increased transmission success is a form of public good. Such strategy might therefore be strongly sensitive to relatedness among co-infecting parasites (see Buckling and Brockhurst, 2008; Lively, 2009, for a discussion on how relatedness affect optimal host exploitation), and to whether costs and benefits from manipulation are equally distributed among coinfecting strains. Under low relatedness, the presence of other strains within a host is expected to lower the per-strain investment in host manipulation because parasites should be less willing to help non-kin (Brown, 1999; Vickery and Poulin, 2010), because of a trade-off in resource allocation under high within-host competition, or because cheating mutants that save on the metabolic cost of manipulation have a selective advantage when co-infecting with manipulative strains (Buckling and Brockhurst, 2008). The first two cases involve a plastic response to co-infection on manipulative effort, whereas the latter one involves an evolutionary response.

A different situation occurs in heterospecific co-infections, when one parasite species which is unable to manipulate the phenotype of its intermediate host’s phenotype benefits from the manipulative effort of another parasite species, with which it shares a common final host. Thomas et al. (1997) coined the term “hitchhiking” to account for the possibility that a non-manipulative species develops an ability to differentially infest hosts already infected by a manipulative one. To our knowledge, the evolutionary dynamics of such a situation has not been investigated so far. One prediction, however, is that hitchhiking should result in a non-random association between the manipulative species and the hitchhiking one among hosts.

A potential for conflict between co-occurring parasites can exist under different circumstances. First, parasites of the same species may have different interests in transmission in terms of timing. It has long been theoretically predicted and experimentally demonstrated that adaptive manipulation enhances transmission specifically when the developmental stage infective to the next host has been reached by the parasite (Parker et al., 2009: manipulation by “predation enhancement”). More recently, the selective advantage of manipulating the host in ways protecting the parasite from premature transmission until infective to the next host has been acknowledged (manipulation in the form of “predation suppression,” Parker et al., 2009). It has thus been predicted that opposite interests between developmental stages should translate into conflict over host manipulation when sequential infection with conspecific parasites occurs (Parker et al., 2009). Given that non-specific predation suppression may evolve more easily than predation enhancement (Parker et al., 2009), and that non-infective stages have more to lose from premature trophic transmission (dead-end) in co-infection than the manipulative infectious stage has from delayed manipulation (time), non-infectious stage are expected to win the conflict over the timing of manipulation.

Second, conflict may occur between trophically transmitted parasites that rely on different definitive hosts to complete their life cycles, and, hence, are not compatible, when at least one of the two is a manipulative parasite. In such a case, different outcomes can be predicted. Conflict of interest may lead parasites species to avoid hosts already infected by a non-compatible parasite species (Lafferty, 1999). This would result in a lower rate of co-infections between non-compatible parasites than expected by chance. Alternatively, one parasite species may out-compete the other and manage to impose its own interest in transmission. For instance, a non-manipulative parasite might be able to prevent its host from being manipulated by a non-compatible parasite, such that co-infected host resume to normal phenotype. In the case of a conflict between two non-compatible, manipulative parasites, the host would express the altered phenotype induced by the most competitive parasite. Finally, non-compatible manipulative parasites might be unable to counteract each other, resulting in mixed altered phenotype in the host.

Third, conflict may oppose parasites with horizontal transmission to parasites with vertical one, when the former has a negative impact on the reproductive output of their common host. Total or partial castration of the intermediate host by a manipulative macroparasite would clearly be detrimental to the fitness of vertically transmitted microparasites, such that natural selection may have favored in the latest the ability to counteract castration, fully or partially.

## INTERACTIONS BETWEEN CO-OCCURING PARASITES: EMPIRICAL EVIDENCE

### EVIDENCE FOR COOPERATION BETWEEN CONSPECIFIC PARASITES INFECTING A SINGLE HOST

Only a few studies so far have examined in detail the effect of multiple infections by a single parasite species on host manipulation. Using naturally infected *G. pulex*, Cézilly et al., (2000) observed no change in altered behavior with the intensity of infection with either *Pomphorhynchus laevis* or the bird acanthocephalan *Polymorphus minutus*. Conversely, using experimental infections of *G. pulex* by *Pomphorhynchus laevis*, Franceschi et al. (2008) found that manipulation (reversed phototaxis) was higher in hosts infected with two parasites than in singly infected ones, with no further increase in manipulation at higher intensities. In addition, using the same host-parasite association, Dianne et al. (2012) observed that all co-infecting parasites did not equally suffer from intraspecific competition. Larval size was positively correlated with host phototaxis in single-infected individuals, but not at higher infection intensities, possibly because competition for host resources affects larval growth and manipulative abilities of co-infecting larval acanthocephalans.

Host-parasite associations where the investment into manipulation is associated with a specific localization in the host provide better opportunities to evaluate the potential for sysnergistic interactions at the intraspecific level. For instance, metacercariae of the trematode *Curtuteria australis* (Echinostomatidae) all encyst in the foot muscle of their host, the New Zealand cockle, *Austrovenus stutchburyi*, but only those localized at the tip of the foot can impair borrowing behavior, hence increasing trophic transmission to avian final hosts. This manipulative strategy comes at a cost since fish cropping of the foot tip decreases the chances of survival before getting transmitted of individuals encysted there (Leung et al., 2010). Surprisingly, newly arriving cercariae encyst in the foot tip of already infected cockles, instead of taking the opportunity of a safer location without loosing the benefits from impaired burrowing on transmission success. This apparent cooperation in manipulative effort may arise due to the necessity to reach a minimum threshold number of cercariae to significantly impair burrowing (Leung et al., 2010).

### CONFLICT BETWEEN DEVELOPMENTAL STAGES OVER THE TIMING OF TRANSMISSION

Several studies of the phenotypic alterations brought about by trophically transmitted parasites have provided evidence for a switch in the behavior of infected intermediate hosts from reduced exposition to predation to increased one over the course of the parasite’s development (Hammerschmidt et al., 2009; Dianne et al., 2011; Weinreich et al., 2013). A similar phenomenon has been reported in vector-born malaria parasites, with opposite effects of the infectious stage (sporozoïte) and the pre-infectious stage (oocyst) on the behavior of mosquitoes, more specifically on blood-feeding and other risky behaviors (several references in Cator et al., 2012, 2013).

However, to our knowledge, only two studies have examined the evidence in favor of a conflict over the timing of transmission. Sparkes et al. (2004) examined the potential for such a conflict in the acanthocephalan parasite *Acanthocephalus dirus* that is known to induce a color change, from dark to light-colored, in its intermediate host, the aquatic isopod *Caecidotea intermedius*, supposed to increase exposure to predation by fish final hosts (Camp and Huizinga, 1979). Non-infective stages (acanthella) of the parasite induce a color change over about 40% of the host’s body, whereas infective ones induce a color change above 80%. Despite a potential for conflict over the extent of color change, Sparkes et al. (2004) found that hosts co-infected with infective and non-infective stages were similar in the degree of color change to hosts infected by an infective stage, contrary to theoretical predictions (Parker et al., 2009). On the other hand, using both field data and experimental infections, Dianne et al. (2010) have shown that the presence *Pomphorhynchus laevis* acanthella delays the reversal of phototaxis induced by cystacanths (mature parasites), without however suppressing it, resulting in an intermediate level of manipulation when co-infection associates the two larval stages.

### CO-INFECTIONS WITH HETEROSPECIFIC PARASITES

Multiple infections between heterospecific parasites are commonly observed in the wild, and, in some cases, occur more often than by chance, possibly because variation exists between hosts in immunocomptence and, hence, susceptibility to infection (Poulin, 2007). However, detailed studies on interspecific interactions involving at least one manipulative parasite are few.

In particular, evidence for hitch-hiking between a non-manipulative parasite and a manipulative one remains limited. According to Thomas et al. (1997), the trematode *Maritrema subdolum* increases its own transmission to avian final hosts through preferentially infecting *Gammarus insensibilis* already infected by the trematode *Microphallus papillorobustus.* The latter appears to enhance its transmission to aquatic birds by inducing in their amphipod hosts a positive phototaxis, a negative geotaxis and an aberrant evasive behavior, which presumably make them more susceptible to predation by aquatic birds (Helluy, 1984). Metacercariae of *M. subdolum* were positively associated with those of *M. papillorobustus* among amphipod hosts in the field. In addition, laboratory experiments showed that cercariae of *M. subdolum* actively swam towards the top of the water column, then increasing their probability of encountering amphipods already infected by *M. papillorobustus*. However, Mouritsen (2001) contended that cercariae of *M. subdolum* actually do not swim in the water column, but instead crawl at the sediment–water interface, and concluded that *M. subdolum* cannot be a hitch-hiker as suggested by Thomas et al. (1997), whereas a likely candidate would be another species of *Microphallus*. Altenatively, the difference between the two studies might be due to the fact that the behavior of the cercariae of *M. subdolum* is different in different regions (Thomas and Helluy, 2002), although the reason for such a difference remains obscure.

A similar case of non-random association between a non-manipulative parasite and a manipulative one has been investigated by Leung and Poulin (2007a, 2007b). It consists in a positive association between infection intensity of the metacercariae of foot-encysting echinostomes and that of gymnophallid metacercariae in their common host, the cockle, *Austrovenus stutchburyi*. The authors first suggested that the gymnophallid was a hitch-hiker parasite because, in addition to the pattern of positive association, it shares the same transmission route as the echinostomes, but unlike the echinostomes, it is unable to manipulate the burrowing behavior of cockles, and increase its transmission to avian final hosts (Leung and Poulin, 2007a). To test this hypothesis, they conducted a field experiment involving cockles forced to remain either above or below the sediment surface to simulate manipulated and non-manipulated hosts. There was however no evidence for a preference of gymnophallids for either surfaced or buried cockles, thus refuting the hitchiking hypothesis (Leung and Poulin, 2007b).

Evidence for synergistic efforts in manipulation between different parasite species remains scarce, too. Poulin et al. (2003) investigated interactions among three helminth species, one acanthocephalan, one trematode and one nematode, co-occurring in two species of crab as intermediate hosts and exploiting shorebirds as final hosts. They found no measurable effect of other helminth species on the size of acanthocephalans, suggesting no interspecific conflict over resource use within crabs. However, concentrations of serotonin (a neuromodulator involved in the altered phototaxis of crustaceans hosts infected with acanthocephalans and trematodes, Helluy and Thomas, 2003; Tain et al., 2006, 2007) in the brains of one crab species were negatively related to the numbers of acanthocephalans and trematodes, but not to nematodes, suggesting of a potentially synergistic manipulation of host behavior by the two helminth species.

Although conflict in manipulation seems obvious when manipulative parasites targeting contrasted species as final hosts co-occur in a single intermediate host, only a few studies have directly assessed the outcome of such interactions. Cézilly et al. (2000) examined the alteration of both phototaxis and geotaxis in *G. pulex* co-infected by *Pomphorhynchus laevis* and *Polymorphus minutus* and found that outcome of the antagonism between the two parasites differed between the two traits. One the one hand, reversed geotaxis (inducing hosts to swim closer to the surface where they presumably become more exposed to bird predators) of gammarids harboring both parasites was less pronounced than that of individuals infected with only *Polymorphus minutus*. On the other hand, altered phototaxis of individuals infected with both parasites was similar to that of individuals infected with only *Pomphorhynchus laevis*. In addition, using large samples collected in the field, Outreman et al. (2002) found no evidence for non-random association between the same two parasite species among their common intermediate hosts.

Looking at interactions between more distantly related parasite species may, however, provide a different perspective. For instance, using naturally infected individuals, Rauque et al. (2011) found that co-infection with the trematode *Microphallus* sp. impaired the ability of both the acanthocephalan *Acanthocephalus galaxii* and a cyclophyllidan cestode to alter the phototaxis of the amphipod *Paracalliope fluviatilis*. However, caution must be exerted when interpreting such results. For instance, Fauchier and Thomas (2001) found a negative association between the non-manipulative nematode *Gammarinema gammari* and *M. papillorobustus* among male *G. insensibilis* in the field, suggestive of a conflict of interest between the two parasites. Indeed, Thomas et al. (2002) showed that among amphipods naturally infected by the trematode, those who did not display altered phototaxis also had more nematodes, suggesting that the nematode could cancel the manipulation effort of the trematode, and hence avoid a premature death. However, altered phototaxis was maintained after exposing manipulated amphipods to nematodes, while the experimental elimination of nematodes from non-manipulated co-infected amphipods did not restore manipulation.

### HORIZONTAL TRANSMISSION, VIRULENCE AND CASTRATION VS. VERTICAL TRANSMISSION

Among multiple parasite infections, one case deserves particular attention because of its strong potential source of conflict: the co-infection by manipulative parasites and vertically transmitted (VT) parasites. There is now growing evidence that VT, maternally transmitted, microorganisms are ubiquitous in invertebrates (Vautrin and Vavre, 2009). In all cases, vertical transmission necessitates survival of the “vector” host, i.e., the mother. As noted earlier, most manipulative parasites increase host mortality. Trophically transmitted parasites are of particular interest because, as for parasitoids, their transmission critically relies on host death. Indeed, their transmission to the definitive host implies the death of the intermediate host by predation, a phenomenon that can be seen as an extreme example of virulence (Poulin and Combes, 1999). VT parasites or symbionts often reach high prevalence in hosts’ populations. At the individual level, they are present in a given host individual at its birth, i.e., before any other super-infection by a horizontally-transmitted (HT) parasite. Therefore, provided the risk of infection by a virulent HT parasite is not too low, natural selection should potentially favors VT parasite variants able to fight either the presence or the effects of HT virulent parasites (Rigaud and Haine, 2005).

Several studies are now available showing that such symbiont-mediated protection (Haine, 2008) have been selected against parasitoids or other types of parasites lethal to the host (e.g., Rouchet and Vorburger, 2012; Lukasik et al., 2013, respectively, for recent examples). Models on the ecology and evolution of this type of conflict between VT and HT parasites revealed that protection is more likely to evolve (i) in long-living hosts, (ii) in response to HT that causes a significant reduction in host fecundity (e.g., those castrating their hosts), but inducing moderate levels of virulence (Jones et al., 2011). However, protective symbionts are typically found in only a fraction of the host population, a phenomenon that could be due to a very fine balance between the costs induced by symbionts and the strength of protection they provide (Kwiatkowski and Vorburger, 2012).

Only a few studies testing specifically the conflicts between VT and manipulative parasites are available. One study tested the effect of a VT, feminizing, microsporidium (*Dictyocoela* sp.) on the outcome of co-infections with acanthocephalans in their *G. roeseli* hosts (Haine et al., 2005). The VT parasites did not protect their hosts against acanthocephalan infections, but reduces the geotaxis inversion induced by *Polymorphus minutus* (a behavioral change increasing the probability of predation by the bird definitive host). This ”sabotage” of behavioral change occurs against the acanthocephalan species that, in addition, castrate 75% of female hosts. Sabotage was not found against *Pomphorhynchus laevis*, another acanthocephalan species inducing a moderate reduction in fecundity (Haine et al., 2005), fitting the prediction of Jones et al. (2011) that VT parasites could more easily select for resistance against a castrating parasite. However, the microsporidia did not influence the degree of castration among females infected by *Polymorphus minutus.* A second study explored the effect of the partial VT microsporidian parasite *Octosporea bayeri* (also using horizontal transmission in its life-cycle) when occurring in co-infections with the HT blood-infecting, castrating, bacterium *Pasteuria ramosa* in *Daphnia* hosts (Ben-Ami et al., 2011). When the two parasites co-infect the hosts after both using horizontal transmission, *Pasteuria* competitively excluded *Octosporea*, and characteristics of double infections resembled those of single infection by *Pasteuria*. When hosts became first vertically (transovarilly) infected with *Octosporea*, there was no evidence that the VT parasite protects its host against *Pasteuria*, neither in protecting from co-infection nor in avoiding castration. However, *Octosporea* was able to withstand competition with *Pasteuria* to some degree, and was able to produce infective stages. However, both parasite species suffer from the co-infections (i.e., produced less infective stages than in single infections) and co-infections led to the expression of higher virulence, a probable consequence of intra-host parasite competition.

These two examples differ in many characteristics of both hosts and parasites life-histories. The differences in the outcome of multiple infections between VT and HT parasites are therefore not surprising. However, it is worth noting that acanthocephalan/microsporidia multiple infections in *Gammaru*s *roeseli* are not rare in the wild (see also Gismondi et al., 2012), while *Pasteuria*/*Octospora* multiple infections in *Daphnia* are much rarer in natural populations (Ebert et al., 2001). The differences in strength of the selective pressure exerted by the HT parasites, i.e., in the prevalence of multiple infections, might therefore explain why in one case host protection by “sabotage” has been selected in VT parasites and not in the other example.

## CONCLUSION AND PERSPECTIVES

Although, theoretical considerations suggest that interactions between parasites within a single host may shape manipulation, the available empirical evidence, at least concerning interactions between macroparasites in invertebrate hosts, is surprisingly thin. This may be due to several reasons. First, although appealing, predictions about a plastic adjustment or an evolutionary response of individual manipulative effort to co-infection are difficult to test experimentally. Indeed, in most host-parasite systems, individual manipulative effort can be only indirectly estimated from its consequence on the parasite’s fitness in the next host, such that potential trade-offs between manipulation of intermediate host and survival and reproductive success within the final host are most often difficult to quantify. Second, competition rather than cooperation might the dominant force in infra-populations of manipulative parasites, if, for instance, parasites that are co-occurring in a single intermediate host suffer from reduced size and fecundity (see for instance Fredensborg and Poulin, 2005). Third, the potential conflict of interest between co-occurring manipulative parasites might be reduced if there is a high risk of dying within the intermediate host before reaching a definitive host. In such a case, reducing such a risk at the expenses of specificity in transmission may be favored by natural selection (see Seppälä and Jokela, 2008; Cézilly et al., 2010). Fourth, it has been suggested that given the relatively low frequency of manipulative parasites, co-occurrence is generally a very rare event in the field. Consequently, such a weak selection pressure should have no important consequences beyond the host individual level (Rauque et al., 2011). However, this might not be a correct way of reasoning. Let’s consider the hypothetical case of two manipulative parasite species, A and B, exploiting in sympatry a common intermediate host but depending on different species as final hosts, and, hence, inducing contrasted phenotypic alterations. Let’s assume that the prevalence of parasite A is 5%, whereas that of parasite B is 20%. In the absence of non-random association, the expected prevalence of mixed infections among intermediate hosts would be as low as 1%. Still, any parasite A would still have a 20% chance of infesting a host already infected with parasite B, a rate that might be sufficient to favor the evolution of an ability for parasite A to overpower parasite B. Note that the symmetrical selective pressure would be less for parasite B, as its chance of co-occurring with parasite A when infecting an intermediate host is only 5%. This simple example suggests that the outcome of interactions between parasites may also depend on their relative prevalence within the intermediate host population.

Clearly, the study of the consequences of both monospecific and plurispecific co-infections by parasites on host phenotype deserves further consideration, both on theoretical and empirical grounds. In particular, more attention should be given about the importance of relatedness between parasites on the outcome of co-occurrence within a single host (Brown, 1999; see Keeney et al., 2007). In that respect, recent progress with experimental infections combined with the use of refined molecular tools to assess genetic relatedness may prove useful in the future.

However, we would like to emphasize here the neglected importance of micro-organisms in the study of host manipulation by parasites. Clearly, potential conflict between vertically transmitted micro-organisms and macro-parasites inducing total or partial castration offers interesting perspectives for future research. More generally, the relevance of microorganisms for the study of host manipulation by parasites deserves further attention. Microorganisms are ubiquitous and can have important, although subtle effects on their hosts’ phenotype. For instance, it is now accepted that variation in the composition of several microbiomes (gut or skin microbiota for example) can alter the behavior of various species (fruitflies, mosquitoes, or mice; reviewed in Ezenwa et al., 2012). These microbiomes are ubiquitous in animals, and almost nothing is known on their interaction with other “passengers” of their hosts, in particular manipulative parasites. Such interactions could be direct if the microbiomes influence the manipulative effects of parasites (possibly through the behavioral changes they induce themselves), or indirect, through the regulation of the host’s immune system. For instance, it has been recently shown that non-pathogenic aquatic bacteria can activate the immune system and increase predation risk of *Enallagma cyathigerum* damselfly larvae (Janssens and Stoks, 2014). To what extent this result extends to other invertebrate species, and particularly to species that are exploited as intermediate hosts by macro-parasites with complex life cycle, remains to be assessed. So far, studies of the phenotypic alterations induced by macro-parasites have not considered the possibility that hosts would be simultaneously infected by microorganisms, although this is very likely when studying naturally infected hosts collected in the field. Because of their reduced immunocompetence, hosts infected with certain micro-organisms, pathogenic or non-pathogenic, might be more likely to become infected by macroparasites (Cornet et al., 2009), opening the possibility that the observed, and often non-specific (see Kaldonski et al., 2008), increased vulnerability to predators of intermediate hosts infected with manipulative macro-parasites is a more complex phenomenon than previously thought. How much speculative such considerations can be, they strongly argue in favor of an increased interest in the role of micro-organisms in the phenomenon of host manipulation by parasites.

## Conflict of Interest Statement

The authors declare that the research was conducted in the absence of any commercial or financial relationships that could be construed as a potential conflict of interest.
